# Supplementary material for: Unveiling the Bioactive Potential of the Invasive Jellyfish Phyllorhiza punctata Through Integrative Transcriptomic and Proteomic Analyses
Source: Biomolecules. 2025 Aug 4;15(8):1121. doi: 10.3390/biom15081121 (PMC12383608; doi:10.3390/biom15081121)
Supplement: Supplementary file 1 [file biomolecules-15-01121-s001.zip › Suplementary Material/FileS3/PEPFOLD4/XP_065068864_13072/clusters.html]

 PEP-FOLD clusters


Download archive of all models  here   

|  |  |  |
| --- | --- | --- |
| # | file | sOPEP |
| # cluster 1 - Size: 6 |  |  |
|  | XP\_065068864\_13072-model1 | -13.6436 |
|  | XP\_065068864\_13072-model1.1 | -13.5224 |
|  | XP\_065068864\_13072-model1.2 | -13.198 |
|  | XP\_065068864\_13072-model1.3 | -12.952 |
|  | XP\_065068864\_13072-model1.4 | -12.8557 |
|  | XP\_065068864\_13072-model1.5 | -12.6805 |
| # cluster 2 - Size: 5 |  |  |
|  | XP\_065068864\_13072-model2 | -13.5568 |
|  | XP\_065068864\_13072-model2.1 | -13.3843 |
|  | XP\_065068864\_13072-model2.2 | -13.2864 |
|  | XP\_065068864\_13072-model2.3 | -13.2769 |
|  | XP\_065068864\_13072-model2.4 | -11.6005 |
| # cluster 3 - Size: 9 |  |  |
|  | XP\_065068864\_13072-model3 | -10.7183 |
|  | XP\_065068864\_13072-model3.1 | -10.6665 |
|  | XP\_065068864\_13072-model3.2 | -9.67984 |
|  | XP\_065068864\_13072-model3.3 | -9.67693 |
|  | XP\_065068864\_13072-model3.4 | -9.54993 |
|  | XP\_065068864\_13072-model3.5 | -9.3408 |
|  | XP\_065068864\_13072-model3.6 | -9.18972 |
|  | XP\_065068864\_13072-model3.7 | -9.08143 |
|  | XP\_065068864\_13072-model3.8 | -8.64441 |
| # cluster 4 - Size: 7 |  |  |
|  | XP\_065068864\_13072-model4 | -10.6611 |
|  | XP\_065068864\_13072-model4.1 | -10.3815 |
|  | XP\_065068864\_13072-model4.2 | -10.1169 |
|  | XP\_065068864\_13072-model4.3 | -10.107 |
|  | XP\_065068864\_13072-model4.4 | -10.0007 |
|  | XP\_065068864\_13072-model4.5 | -9.87184 |
|  | XP\_065068864\_13072-model4.6 | -9.15095 |
| # cluster 5 - Size: 7 |  |  |
|  | XP\_065068864\_13072-model5 | -10.5415 |
|  | XP\_065068864\_13072-model5.1 | -10.0096 |
|  | XP\_065068864\_13072-model5.2 | -9.97478 |
|  | XP\_065068864\_13072-model5.3 | -9.84308 |
|  | XP\_065068864\_13072-model5.4 | -9.7852 |
|  | XP\_065068864\_13072-model5.5 | -9.62022 |
|  | XP\_065068864\_13072-model5.6 | -9.05691 |
| # cluster 6 - Size: 9 |  |  |
|  | XP\_065068864\_13072-model6 | -10.5188 |
|  | XP\_065068864\_13072-model6.1 | -10.4527 |
|  | XP\_065068864\_13072-model6.2 | -10.4377 |
|  | XP\_065068864\_13072-model6.3 | -10.2085 |
|  | XP\_065068864\_13072-model6.4 | -10.1871 |
|  | XP\_065068864\_13072-model6.5 | -10.1348 |
|  | XP\_065068864\_13072-model6.6 | -9.53367 |
|  | XP\_065068864\_13072-model6.7 | -9.32782 |
|  | XP\_065068864\_13072-model6.8 | -8.90939 |
| # cluster 7 - Size: 4 |  |  |
|  | XP\_065068864\_13072-model7 | -10.4611 |
|  | XP\_065068864\_13072-model7.1 | -9.86449 |
|  | XP\_065068864\_13072-model7.2 | -9.70425 |
|  | XP\_065068864\_13072-model7.3 | -9.26837 |
| # cluster 8 - Size: 3 |  |  |
|  | XP\_065068864\_13072-model8 | -10.3514 |
|  | XP\_065068864\_13072-model8.1 | -9.55681 |
|  | XP\_065068864\_13072-model8.2 | -9.14401 |
| # cluster 9 - Size: 5 |  |  |
|  | XP\_065068864\_13072-model9 | -10.3483 |
|  | XP\_065068864\_13072-model9.1 | -9.78293 |
|  | XP\_065068864\_13072-model9.2 | -9.16849 |
|  | XP\_065068864\_13072-model9.3 | -8.84469 |
|  | XP\_065068864\_13072-model9.4 | -8.52202 |
| # cluster 10 - Size: 6 |  |  |
|  | XP\_065068864\_13072-model10 | -10.3111 |
|  | XP\_065068864\_13072-model10.1 | -9.72082 |
|  | XP\_065068864\_13072-model10.2 | -9.33175 |
|  | XP\_065068864\_13072-model10.3 | -9.18737 |
|  | XP\_065068864\_13072-model10.4 | -9.10294 |
|  | XP\_065068864\_13072-model10.5 | -8.86925 |
| # cluster 11 - Size: 1 |  |  |
|  | XP\_065068864\_13072-model11 | -10.265 |
| # cluster 12 - Size: 4 |  |  |
|  | XP\_065068864\_13072-model12 | -9.94492 |
|  | XP\_065068864\_13072-model12.1 | -9.8016 |
|  | XP\_065068864\_13072-model12.2 | -9.02759 |
|  | XP\_065068864\_13072-model12.3 | -8.01509 |
| # cluster 13 - Size: 1 |  |  |
|  | XP\_065068864\_13072-model13 | -9.92058 |
| # cluster 14 - Size: 1 |  |  |
|  | XP\_065068864\_13072-model14 | -9.85974 |
| # cluster 15 - Size: 1 |  |  |
|  | XP\_065068864\_13072-model15 | -9.69335 |
| # cluster 16 - Size: 8 |  |  |
|  | XP\_065068864\_13072-model16 | -9.50642 |
|  | XP\_065068864\_13072-model16.1 | -9.50642 |
|  | XP\_065068864\_13072-model16.2 | -9.39098 |
|  | XP\_065068864\_13072-model16.3 | -9.2817 |
|  | XP\_065068864\_13072-model16.4 | -9.15113 |
|  | XP\_065068864\_13072-model16.5 | -8.91297 |
|  | XP\_065068864\_13072-model16.6 | -8.7677 |
|  | XP\_065068864\_13072-model16.7 | -8.7136 |
| # cluster 17 - Size: 11 |  |  |
|  | XP\_065068864\_13072-model17 | -9.31244 |
|  | XP\_065068864\_13072-model17.1 | -9.25713 |
|  | XP\_065068864\_13072-model17.2 | -9.21722 |
|  | XP\_065068864\_13072-model17.3 | -9.13707 |
|  | XP\_065068864\_13072-model17.4 | -9.13077 |
|  | XP\_065068864\_13072-model17.5 | -9.04344 |
|  | XP\_065068864\_13072-model17.6 | -8.99415 |
|  | XP\_065068864\_13072-model17.7 | -8.9243 |
|  | XP\_065068864\_13072-model17.8 | -8.86269 |
|  | XP\_065068864\_13072-model17.9 | -8.76155 |
|  | XP\_065068864\_13072-model17.10 | -8.65812 |
| # cluster 18 - Size: 2 |  |  |
|  | XP\_065068864\_13072-model18 | -9.16098 |
|  | XP\_065068864\_13072-model18.1 | -9.03587 |
| # cluster 19 - Size: 2 |  |  |
|  | XP\_065068864\_13072-model19 | -9.14304 |
|  | XP\_065068864\_13072-model19.1 | -8.97417 |
| # cluster 20 - Size: 1 |  |  |
|  | XP\_065068864\_13072-model20 | -8.99274 |
| # cluster 21 - Size: 4 |  |  |
|  | XP\_065068864\_13072-model21 | -8.95883 |
|  | XP\_065068864\_13072-model21.1 | -8.87442 |
|  | XP\_065068864\_13072-model21.2 | -8.75389 |
|  | XP\_065068864\_13072-model21.3 | -8.56736 |
| # cluster 22 - Size: 1 |  |  |
|  | XP\_065068864\_13072-model22 | -8.94737 |
| # cluster 23 - Size: 1 |  |  |
|  | XP\_065068864\_13072-model23 | -8.51182 |
| # cluster 24 - Size: 1 |  |  |
|  | XP\_065068864\_13072-model24 | -8.40347 |
|  |  |  |
